# Effects of Fetal Bovine Serum deprivation in cell cultures on the production of *Anticarsia gemmatalis *Multinucleopolyhedrovirus

**DOI:** 10.1186/1472-6750-10-68

**Published:** 2010-09-15

**Authors:** Diego L Mengual Gómez, Mariano N Belaich, Vanina A Rodríguez, Pablo D Ghiringhelli

**Affiliations:** 1 Laboratorio de Ingeniería Genética y Biología Celular y Molecular, Departamento de Ciencia y Tecnología, Universidad Nacional de Quilmes Roque Saenz Peña 352, CP B1876BXD Bernal, Argentina

## Abstract

**Background:**

*Anticarsia gemmatalis *is a pest in South America's soybean crops, which could be controlled by the Multinucleopolyhedrovirus of *A. gemmatalis *(AgMNPV). Currently, its commercial production is based on infected larvae. However, the possibility of using modified baculoviruses in Integrated Pest Management programs has stimulated an interest to develop alternative multiplication processes. This study evaluated the AgMNPV production in UFL-Ag-286 cells previously deprived Fetal Bovine Serum.

**Results:**

Culture media containing 1% FBS during the previous 48 hours achieved a synchronized condition where 90% of cells were found in G_0_/G_1 _stage, showing the presence of non-filamentous actin. All characteristics were estimated from cellular viability tests, cell actin detection trials and flow cytometer cell cycle analysis. AgMNPV production was tested by transcript studies and budded viruses (BVs) and occlusion bodies (OBs) yield quantitation. Results showed that the productivity in FBS deprived cells was 9.8 times more in BVs and 3.8 times more in OBs with respect to non-treated cells.

**Conclusions:**

UFL-Ag-286 cells previously deprived in FBS shown to be a better host for AgMNPV propagation, increasing the useful for both *in vitro *bioinsecticide production and applications such as recombinant protein expression or gene delivery.

## Background

Baculoviruses are arthropod-specific viruses containing large double-stranded circular DNA genomes of 80000-180000 bps. A characteristic of these viruses is the presence of two different phenotypes during virus infection: budded virus (BVs) in the initial part of multiplication cycle and occlusion bodies (OBs) at the end of replication [1,2]. In nature, primary infection takes place in the insect midgut cells after ingestion of OBs, and then the initial progeny of BVs is responsible for systemic infection [3,4]. Finally, the OBs are produced during the last phase of cycle and comprise virions embedded in a protein matrix that protects the viruses in the environment [5,6]. Baculoviruses have been used extensively in many biological applications such as protein expression systems, as models of genetic regulatory networks and genome evolution, as putative non-human viral vectors for gene delivery, and as biological control agents against insect pests [7-11]. In particular, the *Anticarsia gemmatalis *Multinucleopolyhedrovirus (AgMNPV) was studied thirty years ago and it is suggested as the most successful example of baculovirus applied as bioinsecticide [12-16]. AgMNPV is used to control the velvet bean caterpillar *Anticarsia gemmatalis *Hübner (Lepidoptera: Noctuidae), one of the most important insect pests of soybean crops in America [17].

Usually, the easiest way to replicate baculoviruses is by infection of susceptible larvae colonies. However, it is possible to produce baculoviruses *in vitro *in a more simple way due to the selection of insect cell lines, taking into account current limitations such as the accumulation of point mutations in the fp25K locus conducting to a reduction in the yields of polyhedra [18]. The cell line UFL-Ag-286 from embryos of *Anticarsia gemmatalis *was established to produce AgMNPV in laboratory conditions [19]. Nevertheless, viral production levels are not always suitable to baculovirus applications, forcing the use of alternative strategies that involve the use of methods for viral concentration, which increases costs and production times. This situation stimulates the development of new methods oriented to improve the process of infection in cellular cultures to reach better viral yields. Thus, the production of AgMNPV in UFL-Ag-286 cells could be favored, for example, doing the infection in a specific cell cycle stage. However, there is little information about cycle synchronization in insect cell lines. It was described that *Spodoptera frugiperda *(Sf9) cells are affected when treated with conditioned medium; and it was observed that they are arrested in G_2_/M cell stage by AcMNPV infection generating implications in cell proliferation and recombinant protein production [20-24]. The same authors were also evaluated the effects of synchronization in *Trichoplusia ni *(TN-368) insect cell using similar tests [20-24]. On the other hand, there are standardized protocols for synchronization in mammalian cells, such as those based on serum deprivation, contact inhibition or chemical treatments [23,25-28]. Serum deprivation is used widely for synchronizing cells by arresting them in G_0_/G_1 _phase, but it often reduces cell survival and increases DNA fragmentation [28].

Taking into account the cited antecedents, this work describes the optimization of a synchronization process by serum deprivation in UFL-Ag-286 cells and its impact on the productivity of AgMNPV.

## Results and Discussion

### FBS deprivation and cellular growth

**Figure 1:**
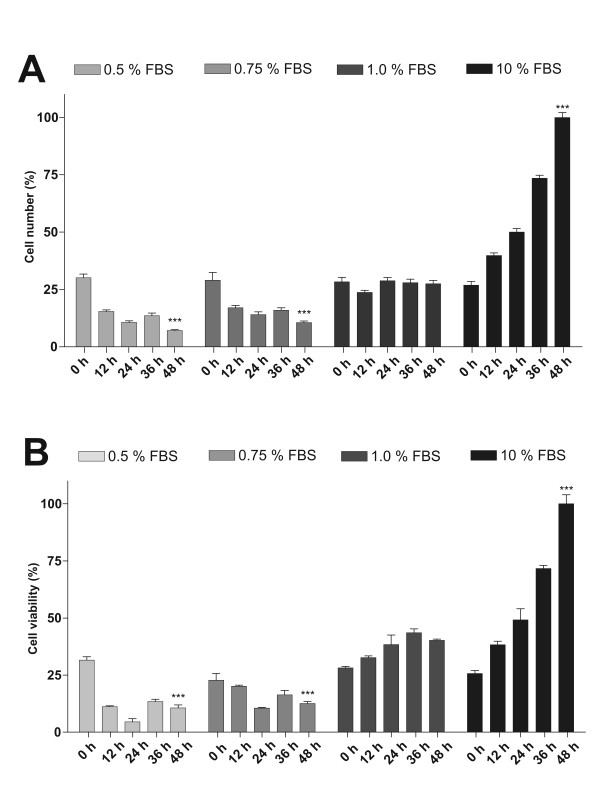
**Growth of UFL-Ag-286 cells in culture conditions with different proportions of FBS**. Monolayers of UFL-Ag-286 cells cultivated in 96 multiwell plates for 48 h were treated with four different concentrations of FBS (0.5, 0.75, 1.0 and 10%) in GRACE's medium. Thereafter, the total number of cells was measured by a colorimetric method **(A)**, and the cell viability was measured by a MTT method **(B)**. For each treatment, data were relativized to the respective 0 h time. Then, the obtained values were normalized with respect to the maximum value. The condition with 1.0% of FBS was not toxic to the cell culture (p = 0.1693, Student's T test), below this concentration the cellular viability was affected (***P < 0.001, *P < 0.05, Student's T test).

In order to obtain synchronized cultures of *Anticarsia gemmatalis *(UFL-Ag 286) cells, different growth conditions in GRACE's medium with decreasing amounts of FBS were tested, because it was reported that the deprivation of this component allows arrest of cell cultures into G_0_/G_1 _cell cycle state [25]. First, with the goal to select the lowest concentration of FBS that did not affect cell survival, the total number of UFL-Ag-286 cells after incubation for 48 h in four different culture conditions (0.5%, 0.75%, 1.0% and 10% of FBS in GRACE's medium) were tested (Figure 1).

The results indicated that cell culture media containing both 0.5% (P < 0.001) and 0.75% (P < 0.05) of FBS caused an unacceptable decrease of the cellular viability, while the treatment with 1.0% FBS allowed to arrest the cell growth and preserve the initial viability (P = 0.1693). It should be noted that cells growing in standard conditions (10% FBS) showed typical behavior of cell multiplication (duplication time about 24-26 h), which is in agreement with previous literature [29]. According to the above, the culture condition of 1% FBS in GRACE's medium was chosen to continue with the trials in comparison with the standard growing condition (10% FBS in GRACE's medium). Lower concentrations of FBS (0.5% and 0.75%) caused a significant decrease in cellular quantity after 48 h of treatment, condition that could induce DNA fragmentation and subsequent cell death [28].

### Cell stages and actin cytoskeleton

**Figure 2:**
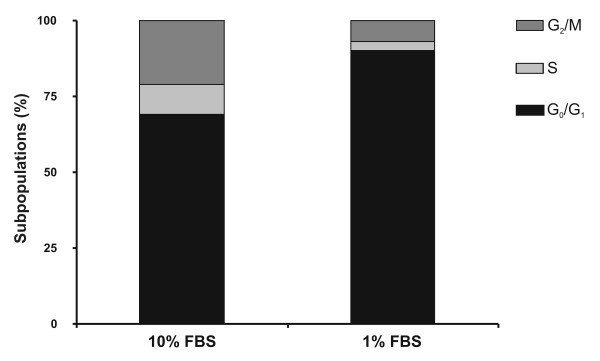
**Subpopulations of UFL-Ag-286 cells**. Monolayers of UFL-Ag-286 cells were treated during 48 h both in standard condition (10% FBS) and synchronized condition (1% FBS) in GRACE's medium, harvested and stained with ethidium bromide, and analyzed by flow cytometer. The bar graph shows the results corresponding to the subpopulation cells according to typical stages (G_2_/M, S and G_0_/G_1_) based on DNA content measures.

Cells growing with 1% FBS or 10% FBS during 48 h were studied by flow cytometer experiments in order to obtain a quantitative determination of cell stage subpopulations. The analysis of *in vitro *cell cultures in active replication state can be achieved by nucleic acid fluorescence labeling and then analyzing the fluorescence properties of each cell in the whole population. Quiescent and G_1 _cells have one unit of nuclear genome and will therefore have 1× fluorescence intensity. On the other hand, cells in phase G_2_/M have two units of nuclear genome and thus have a 2× fluorescence intensity; whereas S phase cells, that are synthesizing DNA, have values of fluorescence intensity intermediate between 1× and 2× (Figure 2). According to this, it was found that after carrying out the deprivation process with 1% FBS in GRACE's medium during 48 h 90% of UFL-Ag-286 cells were in G_0_/G_1_, 3% in S, and 7% in G_2_/M, yielding similar results than those obtained in mammal cells [25-27]. Meanwhile, actively growing cells showed only 69% of the population in G_0_/G_1_, 6% in S and 25% in G_2_/M.

**Figure 3:**
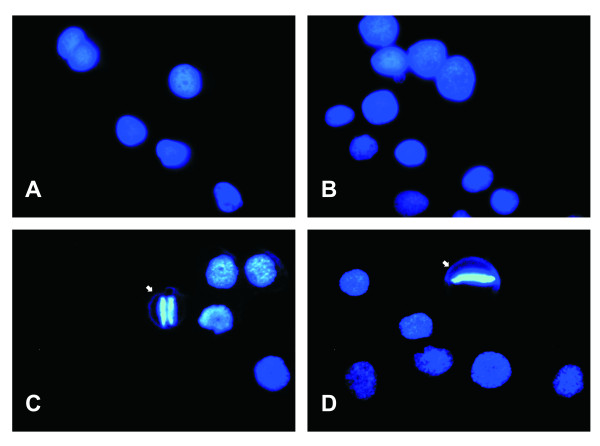
**Microscopic analysis of cell nuclei**. Monolayers of UFL-Ag-286 cells growing in 6 multiwell plates were treated during 48 h both in standard condition (10% FBS) and synchronized condition (1% FBS) in GRACE's medium, and then stained with DAPI. **A **and **B**. Synchronized cells show homogeneous morphology of nuclei in an expanded topology (1000×). **C **and **D**. Non-synchronized cells show heterogeneous morphology with evidence of mitotic events, marked with white arrow (1000×).

**Figure 4:**
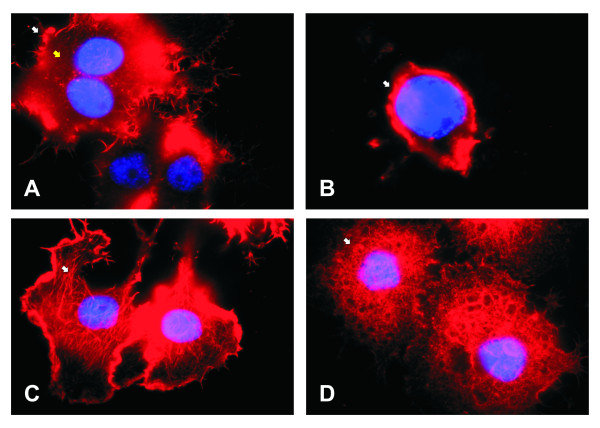
**Microscopic analysis of actin cell cytoskeleton**. Monolayers of UFL-Ag-286 cells growing in 6 multiwell plates were treated during 48 h both in standard condition (10% FBS) and synchronized condition (1% FBS) in GRACE's medium, and then stained with phalloidin-Alexa fluo 355^® ^(red) and DAPI (blue). **A **and **B**. Synchronized cells show diffuse cytoplasm fluorescence, marked with yellow arrows, and actin microfilaments only at the edges, marked with white arrow (1000×). **C **and **D**. Non-synchronized cells show active actin cytoskeleton, marked with white arrows (1000×).

Also, to support that the UFL-Ag-286 cells could be synchronized by serum deprivation in the G_0_/G_1 _phase, stains of nucleus (DAPI) were done. Thus, the images obtained showed nuclei with different morphologies, mitotic events, and evidence of cellular division in cells growing in standard condition. Otherwise, the images for the FBS starved cells showed a more homogenous behavior, with the nuclei in an expanded morphology suggesting a non-mitotic cell cycle state according with flow cytometer results (Figure 3). Moreover, in order to analyze the actin cytoskeleton a specific probe of actin polymers (phalloidin-Alexa fluo 355^®^) was tested in cells growing in both culture conditions (Figure 4). In this way, captured images showed that UFL-Ag-286 cells under standard conditions of growth revealed arrangements of actin, a typical feature of cells in active division. In contrast, serum deprived cells showed mainly diffuse cytoplasm fluorescence, suggesting that cellular actin was in a non-polymerized topology, with just the presence of actin microfilaments associated to the plasma membrane like filopodia.

### Baculovirus production

Once synchronized UFL-Ag-286 cells were in G_0_/G_1 _stage, the productivity of baculovirus infections was tested. First, the virus genome production in BVs was measured. UFL-Ag-286 monolayers, synchronized and non-synchronized, were exposed to AgMNPV-SF (MOI 0.1) and then incubated in GRACE's medium containing 10% FBS during 72 h. The quantitation of viral DNA recovered from BVs from both culture conditions showed that synchronized cells produced 9.4 more viral DNA than non previously FBS deprived cells. Besides, the integrity of genome was confirmed by RFLP assays using HindIII and EcoRI enzymes. An identical pattern of fragments was obtained in both cases (data not shown).

**Figure 5:**
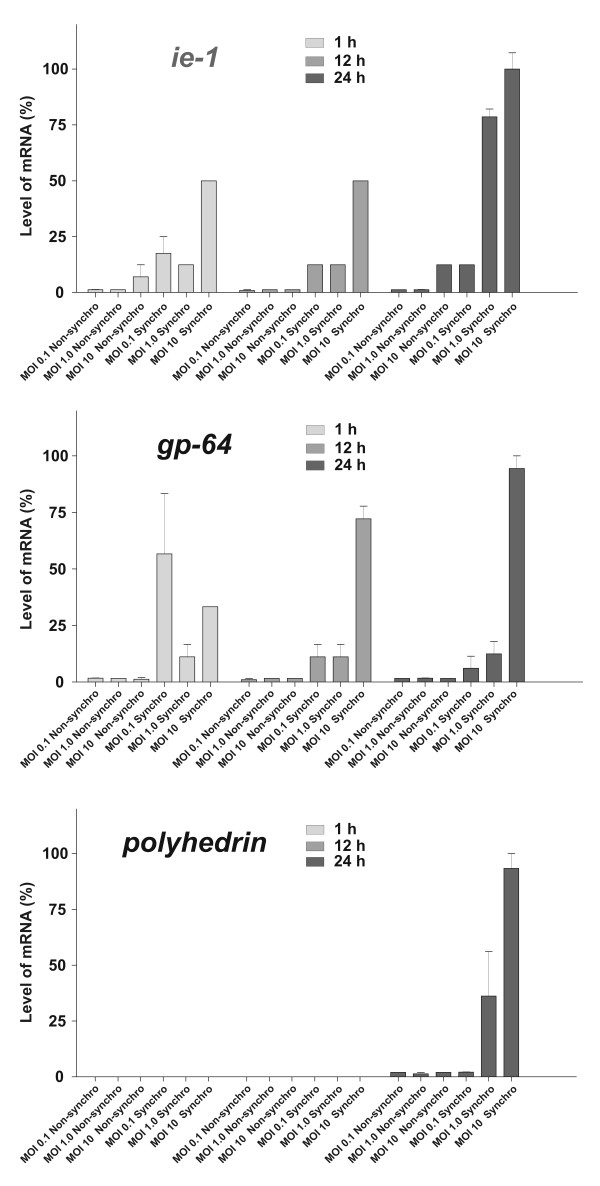
**Transcript analysis of *ie1*, *gp64 *and *polh *genes of AgMNPV**. Monolayers of UFL-Ag-286 cells were treated during 48 h both in standard condition (10% FBS) and synchronized condition (1% FBS) in GRACE's medium, then infected with AgMNPV-SF using three different MOIs (0.1, 1, 10) (n = 3 for each assay); and maintained in GRACE's medium with 10% FBS. After this, cells were collected at 1, 12 and 24 hours to isolate whole RNA, and later hybridized with *ie1*, *gp64*, *polh *(AgMNPV genes) and actin (UFL-Ag-286 gene) probes in independent assays by Slot Blot strategy. The different detected signals for each viral transcript were quantified by image densitometry, relativized using the actin mRNA level and considering as 100% the highest value reached in each test. The error bars are the corresponding standard deviations.

Keeping in mind that the increment in the amount of DNA suggests an increase in the quantity of virions and it should be accompanied by an increment in the viral transcription activity, an mRNA analysis was done. To carry out this, a set of UFL-Ag-286 monolayers previously synchronized by FBS deprivation or growing in standard conditions were exposed to AgMNPV-SF (MOIs: 0.1; 1.0 and 10). First, the mRNA levels of *ie1*, *gp64 *and *polh *genes (taken as markers of different stages of viral cycle) were analyzed with respect to the mRNA level of the cellular *actin *gene by hybridization with specific probes. Actin mRNA was used as reference in transcript analysis because *actin *is one of the housekeeping genes more frequently employed in this kind of studies, together with *alpha-tubuline *or *elongation factor 1 alpha *genes [30,31]. Because there are no sequence information about *Anticarsia gemmatalis*, except for a fragment of *actin *gene (amplified by PCR using universal primers designed *ad hoc *in our laboratory) [15], that sequence was used as an internal standard in mRNA assays. Taking into account that actin transcription could have affected by baculovirus infection processes, the quantity of actin mRNA was estimated along the time period considered in the assays (data not shown). Thus, no significant changes in actin transcription level were observed. In function of this, whole RNAs from infected UFL-Ag-286 cultures in each condition at the three assayed MOIs and in three different times of cycle (1, 12 and 24 h post-infection) were isolated. Then, the relative amounts of each considered gene were tested by Slot Blot hybridization and densitometry analysis (Figure 5).

The transcript analyses showed that synchronized UFL-Ag-286 cells produced 10 to 50 times more specific transcripts than non-synchronized cells. The *ie1 *gene, dependent on cellular RNA polymerase II, was that achieved highest levels of transcription in synchronized cells (1.5 times respect to *gp64 *and 2.3 times respect to *polh *at MOI 1), whereas *gp64 *and *polh *show lowest comparative values probably due to the partial or complete dependency of the viral transcription machinery. It is noteworthy that in the shorter times the behavior was more variable. Even when a synchronized cell culture was used, infections at MOI's 1 or less introduce high variability in the repetitions because the efficiency of primary infection follows a stochastic behavior. This possible effect would become more marked when the observation time was very short (1 h), period in which BVs have not been generated yet and, therefore, the subsequent infections of neighboring cells are not feasible. Secondary infections tend to normalize the situation, leading to infect all cells. However, when the initial MOI was 10, synchronized infection could be assumed, where all susceptible cells could be subject to primo-infection, and initial BVs could not significantly alter the results given the absence of available cells for secondary infection. In spite of these considerations, the fact that the method sensitivity could have affected the obtained results should not be discarded.

**Figure 6:**
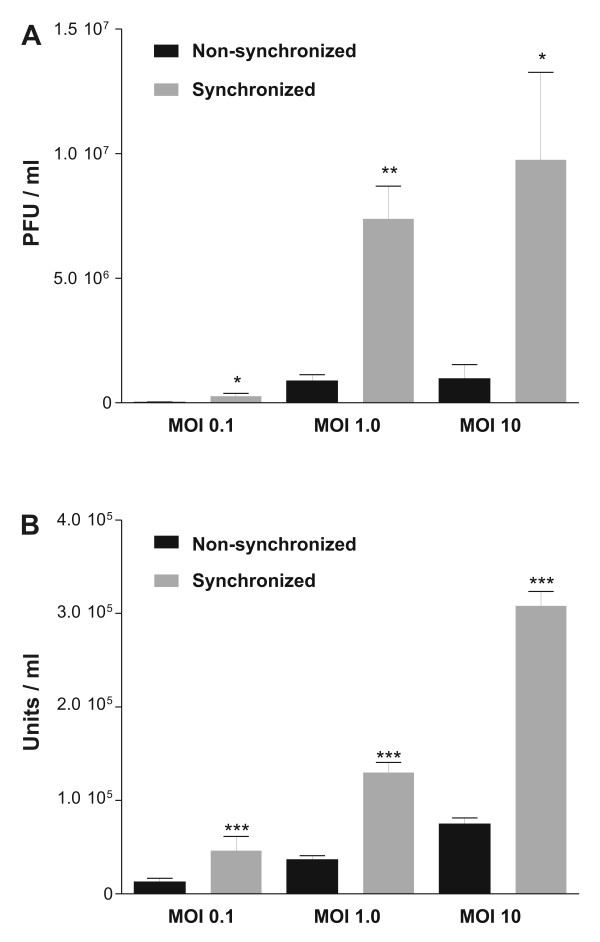
**Baculovirus productivity**. Monolayers of UFL-Ag-286 cells were treated during 48 h both in standard condition (10% FBS, black) or synchronized condition (1% FBS, gray) in GRACE's medium, then infected with AgMNPV-SF using three different MOIs (0.1, 1, 10) (n = 3 for each assay, * P < 0.05, ** P < 0.01, *** P < 0.001, Student's T test); and maintained in GRACE's medium with 10% FBS. **A**. BVs isolated from supernatants and quantified by plaque assays. **B**. OBs isolated from infected cells and quantified by microscopic observation. Error bars are the corresponding standard deviations.

The previous results should be correlated with differences in the production of BVs and OBs. In order to test this, AgMNPV BVs and OBs were isolated from infected UFL-Ag-286 monolayers (MOIs: 0.1, 1 and 10), previously synchronized or non-synchronized. BVs were quantified by titration assays and OBs were quantified by microscopic count (Figure 6).

According to obtained data, an increase in AgMNPV productivity was achieved both in BV and OB phenotypes when cells were previously arrested in G_0_/G_1 _stage. Thus, the AgMNPV infection of synchronized cell cultures produced 9.81 (± 1.15) times of BVs and 3.79 (± 0.30) times of OBs (see Material and Methods) respect to the same assay using cells growing in standard conditions. The increase in virus production was expected because the infection occurs better when cells are in G_1 _stage [32]. However, of the non synchronized cells 69% exhibited the G_0_/G_1 _stage. This could be due to differences in cytoskeleton polymerization/depolymerization induced by the same treatment. Processes of infection at the G_1 _cell stage cause a brief delay in the onset of immediate early virus transcription, while those that occur at other times of cell cycle produce a longer lag phase (double or triple time), because baculoviruses promote cell arrest in G_2 _in order to better exploit the cell machinery in its own multiplication [32]. Another factor that may contribute in increasing the transcription levels and viral production in cells synchronized is the availability of G-actin, because the actin monomers function as transcriptional activators as part of complexes formed by RNApol II and promoter sequences [33]. In fact, there were few actin filaments when UFL-Ag-286 cells were synchronized by serum deprivation, being instead primarily as G-actin. Thus, there would be many more transcription activator complexes and, therefore, a bigger transcriptional activity would be reached [33]. Otherwise, the differences in the achieved increases in the yield of budded and occluded phenotypes between the two conditions tested can be related to the limiting factor given by the accumulation of late and very late proteins in the infected cells. This implies a deep redirection of the cellular biochemical machinery and has a completely requirement of the viral transcriptional machinery.

In addition, and taking into account that it is unknown if the molecular context of the cells is totally homogeneous it should be considered that there were also other modified cellular factors that could have collaborated with the obtained yields.

## Conclusions

Baculoviruses are useful tools for various biological applications, such as heterologous protein expression, as bioinsecticides against agricultural lepidopteran pests or as gene delivery vectors in mammals, among other potential uses [34-36]. For any of these applications is necessary to produce large quantities of viruses, which can be generated *in vivo *using the natural host insect larvae by *per os *infection with OBs; or *in vitro*, using cell lines derived from the same hosts, by infection with BVs. In any case, it is important to optimize production strategies in order to obtain the highest yields of baculoviruses. In particular, we are interested in AgMNPV, the most used baculovirus for biological control of agricultural pests [17,29].

This study has shown that insect cell cultures can be synchronized by serum deprivation, and thereby ensuring that most of them are in G_0_/G_1 _stage. Thus, this cellular sub-phase would be the most susceptible to infection. Moreover, the reduction of actin cytoskeleton and high availability of G-actin would have a positive impact both for the transport of nucleocapsids to the nucleus, and enhancing the transcription of early viral genes.

These results show that the FBS deprivation treatment on UFL-Ag-286 cells during 48 h prior to the infection had a high impact in AgMNPV yields. In conclusion, this method has proven to be useful for increasing the production of AgMNPV in UFL-Ag-286 cells, and also has provided evidence about the influence of host cell phenotype respect to its susceptibility to replicate baculoviruses.

## Methods

### Cells and virus stocks

*Anticarsia gemmatalis *(UFL-Ag-286) cells [18,37] were grown at 27°C in GRACE's medium (Invitrogen, Carlsbad, CA, USA) containing 10% fetal bovine serum (FBS, Bioser, Quilmes, Argentina) and supplied with antibiotics and antimycotics (Invitrogen, Carlsbad, CA, USA). Stocks of AgMNPV-SF [38,39], were grown on monolayers of the UFL-Ag-286 cell line in plastic tissue culture flasks, tittered by plaque assay [40] and maintained as culture supernatants. The UFL-Ag-286 cell line was kindly provided by Bergmann Morais Ribeiro (Laboratorio de Virología e Microscopia Eletrônica, Universidade de Brasilia, Brasil). The AgMNPV-SF initial stock was kindly provided by Juan Daniel Claus (Facultad de Bioquímica y Ciencias Biológicas; Universidad Nacional del Litoral, Argentina).

### FBS deprivation

UFL-Ag-286 cells were grown at 27°C in GRACE's medium containing 0.5%, 0.75%, 1.0% or 10% FBS in 96 multiwell plates. The total number of cells was counted by a colorimetric method at 12, 24, 30, 36 and 48 hours of treatment. Briefly, the medium was removed and the cells were fixed with 10% methanol during 10 minutes and then stained with 0.1% violet crystal. Also, mitochondrial succinate dehydrogenase activity employing a tetrazolium salt, MTT Ultrapure (USB, Cleveland OH, USA), was estimated to study cell viability [41]. Thus, culture medium was replaced by fresh GRACE's medium containing MTT at 0.5 mg/ml. After 3 h incubation, MTT solution was removed and the insoluble formazan crystals were dissolved in 100 μl of dimethylsulfoxide (DMSO, Carlo Erba, Rodano MI, Italy). In both trials absorbance measures were realized at 570 nm in a Dynex Technologies MRX tc Microplate Reader (Sullyfield Circle Chantilly, VA, USA).

### Flow cytometer cell cycle analysis

UFL-Ag-286 cells were cultured and harvested in described conditions, and then distributed in tubes (1 × 10^6 ^cells/tube). The cells were concentrated at 3000 rpm during 10 min, the supernatant was removed and the pellet was resuspended in 150 μl of 1 × PBS. Then, cells were fixed by means of the addition of 350 μl cold ethanol and incubation at -20°C for 1 h. The fixed cells were washed with cold 1 × PBS containing 2% FBS and 0.01% NaN_3 _and were resuspended in 100 μl of 1 × PBS containing 1 mg/ml RNAse (Sigma-Aldrich, Buenos Aires, Argentina). After 30 min at 37°C, 250 μl of 1× PBS supplemented with 50 μg/ml of ethidium bromide and 0.1% Triton X-100 were added. After incubation at 37°C for 1 h, the cells were washed 2 times and resuspended in 1 ml of 1 × PBS with 2% FBS and 0.01% NaN_3_. Flow cytometric studies were performed using a FACS Calibur flow cytometer (Becton Dickinson, San Jose, CA, USA). Cells were analyzed at a rate of 300-500 cells/s using FACS flow (Becton Dickinson) as the sheath fluid and 30000 events were recorded for each sample.

### Fluorescence microscopy of actin cell cytoskeleton

Cell cultures were characterized by fluorescence microscopy in a Nikon Eclipse TE2000, by staining the F-actin with phalloidin-Alexa fluo 355^® ^conjugate and the cell nuclei with DAPI. The reagents were from Molecular Probes (Invitrogen, Carlsbad, CA, USA) and used according to the manufacturer's specifications. To stains, cells at 50% of confluence grown in 12 multiwell plates were fixed with 4% formaldehyde and 1% Triton X-100 (Sigma-Aldrich, Buenos Aires, Argentina) and then treated with 0.165 μM of phalloidin-Alexa fluo 355^® ^during 1 h. After making two washes with 1 × PBS, the cells were spread on slides with mounting medium and DAPI. After excitation with UV-light images were recorded using a Nikon Eclipse TE2000 microscope equipped with a CCD camera.

### Genome and transcript analyses of AgMNPV in cells

UFL-Ag-286 cells, previously grown in GRACE's medium containing 10% FBS or 1% FBS during 48 h in 12 multiwell plates, were exposed to AgMNPV-SF at a multiplicity of infection (MOI) of 0.1 during 1 h in fresh medium without FBS. Once finished that process, media were removed and cells were maintained in GRACE's medium containing 10% FBS. Then, supernatants were recovered at 72 h post-infection and used to isolate BVs and to purify viral DNA by the full scale method [40]. Later, the isolated genomes were quantified by spectroscopy (NanoDrop 1000, Thermo, Wyman Street Waltham, MA, USA).

On the other hand, confluent monolayers of UFL-Ag-286 cells (1 × 10^6 ^cells/ml) were infected with AgMNPV-SF (MOIs 0.1, 1 and 10) under the same conditions previously mentioned, and harvested at 1, 12 and 24 h post infection to analyze the transcription level of *ie1*, *gp64 *and *polh *AgMNPV genes into the viral cycle respect a housekeeping cell gene (*actin*). The RNAs were isolated using Trizol (Invitrogen, Carlsbad, CA, USA) and concentrated by standard alcohol precipitation [42]. After that, RNAs were transferred to a nitrocellulose membrane (Hybond-N+, GE Healthcare Life Sciences, Buenos Aires, Argentina) using a Slot Blot equipment (BIORAD, Alfred Nobel Drive Hercules, CA, USA) and hybridized at 55°C with specific probes for each gene in independent assays. The labeling of probes and the development of the hybridization were made using the AlkPhos Direct System (Genes Images, GE Healthcare Life Sciences, Buenos Aires, Argentina) according to the manufacturer's specifications. Previously to labeling, the specific DNA fragments for each probe were obtained by PCR reactions using Taq DNA pol (PB-L, Quilmes, Argentina) and the following primers [38,43]: *ie1 *with Fwd 5' GATTGTCGGTGAGCGTTGCGCT 3', Rev 5' CATAGAGATTCCGCCA 3'; *gp64 *with Fwd 5' CGCCCAAGGARACGCTG 3', Rev 5' CAAACTTGGTGTTCTCCAT 3'; *polh *with Fwd 5' CGCGGATCCTATGCCAGATTATAG 3', Rev 5' CCCAAGCTTATACGCGGGGCCGGT 3'; *actin *with Fwd 5' GTCCTCTCCCCARTCCGT 3', Rev 5' GTGTGGCGCCTGCTGGGCSTT 3'. The quantitation of hybridization was performed by densitometry analysis (ImagePro Plus Software, Media Cybernetics, Inc., East-West Hwy, Bethesda, MD, USA). Probe calibration using quantified specific templates were carried out to normalize data produced from each transcript determination.

### Baculovirus productivity

UFL-Ag-286 cells at 70% confluence, previously grown in GRACE's medium containing 10% FBS or 1% FBS during 48 h in 12 multiwell plates, were exposed to AgMNPV-SF in three conditions (MOIs: 0.1; 1.0; 10) for 1 h in fresh medium without FBS. Thereafter, media were removed and cells were maintained in GRACE's medium containing 10% FBS. After 48 h incubation, the supernatants were recovered and clarified by centrifugation at 5000 rpm for 10 minutes. The titer of BVs was determined by plaque assay [40]. On the other hand, the cells were harvested in 1 × PBS and then used to isolate the OBs [40], which were quantified by optical microscopic count (Nikon Eclipse TS 1000, Nikon Instruments Inc., Walt Whitman Road, Melville, NY, USA) using Neubauer chamber. To estimate the increasing yields of BV production, the quotients between synchronized and non-synchronized yields for each MOI were calculated. After, a median of all values was obtained. The same calculus was applied for OBs.

## Authors' contributions

DLMG carried out all the experiments, designed the viability and flow cytometric cell cycle tests and drafted the manuscript. MNB designed and carried out the infection assays with their analyses, and drafted the manuscript. VAR collaborated with infection assays and with the preparation of materials needed for all assays. PDG conceived the work, participated in its design and realization, and coordinated the drafting of the manuscript. All authors read and approved the final manuscript.
